# Altered profiles of circulating cytokines in chronic liver diseases (NAFLD/HCC): Impact of the *PNPLA3* ^*I148M*^ risk allele

**DOI:** 10.1097/HC9.0000000000000306

**Published:** 2023-11-22

**Authors:** Mélanie Kirchmeyer, Anthoula Gaigneaux, Florence A. Servais, Anita Arslanow, Markus Casper, Marcin Krawczyk, Frank Lammert, Iris Behrmann

**Affiliations:** 1 Department of Life Sciences and Medicine, University of Luxembourg, Luxembourg; 2 Department of Medicine II, Saarland University Medical Center, Homburg, Germany; 3 Fundació de Recerca Clínic Barcelona-Institut d’Investigacions Biomèdiques August Pi i Sunyer, Barcelona, Spain; 4 Health Sciences, Hannover Medical School MHH, Hannover, Germany

## Abstract

**Background:**

Individuals carrying the risk variant p.I148M of *patatin-like phospholipase domain-containing protein 3* (*PNPLA3*) have a higher susceptibility to fatty liver diseases and associated complications, including HCC, a cancer closely linked to chronic inflammation. Here, we assessed circulating cytokine profiles for patients with chronic liver diseases genotyped for *PNPLA3*.

**Methods:**

Serum concentrations of 22 cytokines were measured by multiplex sandwich-ELISA. The cohort comprised 123 individuals: 67 patients with NAFLD without cirrhosis (57 steatosis, 10 NASH), 24 patients with NAFLD with cirrhosis, 21 patients with HCC (15 cirrhosis), and 11 healthy controls. Receiver operator characteristic analyses were performed to assess the suitability of the cytokine profiles for the prediction of steatosis, cirrhosis, and HCC.

**Results:**

HGF, IL-6, and IL-8 levels were increased in patients, with ∼2-fold higher levels in patients with cirrhosis versus healthy, while platelet derived growth factor-BB (PDGF-BB) and regulated on activation, normal T cell expressed and secreted (RANTES) showed lower concentrations compared to controls. Migration inhibitory factor and monocyte chemoattractant protein-1 (MCP-1) were found at higher levels in NAFLD samples (maximum: NAFLD-cirrhosis) versus healthy controls and HCC samples. In receiver operator characteristic analyses, migration inhibitory factor, IL-8, IL-6, and monocyte chemoattractant protein-1 yielded high sensitivity scores for predicting noncirrhotic NAFLD (vs. healthy). The top combination to predict cirrhosis was HGF plus PDGF-BB. Migration inhibitory factor performed best to discriminate HCC from NAFLD; the addition of monokine induced gamma (MIG), RANTES, IL-4, macrophage colony-stimulating factor (M-CSF), or IL-17A as second parameters further increased the AUC values (> 0.9). No significant impact of the *PNPLA3* ^*I148M*^ allele on cytokine levels was observed in this cohort.

**Conclusions:**

Cytokines have biomarker potential in patients with fatty liver, possibly suited for early HCC detection in patients with fatty liver. Patients carrying the *PNPLA3* risk allele did not present significantly different levels of circulating cytokines.

## Graphical abstract

**Figure:**
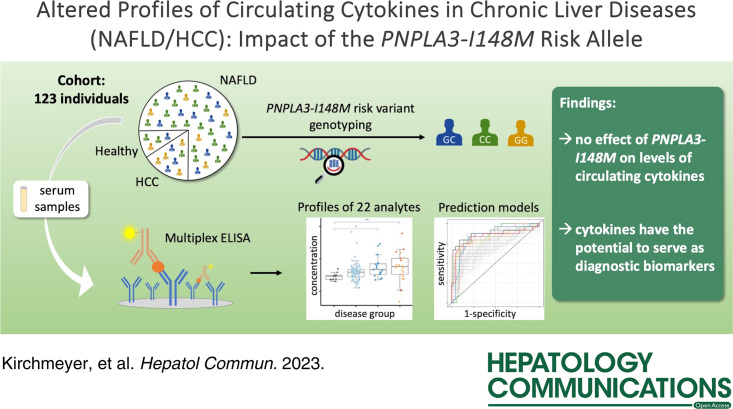

## INTRODUCTION

Liver neoplasms are the third leading cause of cancer-related deaths worldwide, with HCC representing ∼90% of the cases. Chronic hepatic injury, caused by viral hepatitis, alcohol, or toxins, can lead to inflammation through the activation of resident macrophages and stellate cells, followed by hepatocyte necrosis and cell regeneration. These continuous cycles of damage and proliferation foster a chronic liver disease condition that culminates in liver fibrosis and, finally, cirrhosis.^1^

NAFLD is characterized by hepatic fat accumulation in the absence of chronic alcohol consumption and other acute or chronic liver diseases. As the liver manifestation of the metabolic syndrome, fatty liver represents the most common liver disease with a global prevalence of ∼30%.^2^ Simple hepatic steatosis (NAFL) can progress to NASH. This condition is characterized by a combination of steatosis and hepatic inflammation and can lead to liver fibrosis and cirrhosis and an elevated HCC risk. Biomarkers to identify patients at risk would be very useful, considering that NAFLD represents the fastest-growing cause of HCC in many parts of the world.^2,3^

The patatin-like phospholipase domain-containing protein 3 (PNPLA3) is a lipase that also has acyl-transferase activity. PNPLA3 is predominantly expressed in the liver, particularly in hepatocytes and HSC. The *PNPLA3* rs738409 p.I148M minor allele accounts to a large extent for the heritability of NAFLD diseases, as first shown in a genome-wide association study regarding the susceptibility to NAFLD and hepatic fat accumulation. Thereafter, a strong association of this risk allele, present in a high percentage of individuals (eg, ∼40%–50% in Europeans), was also found for other liver diseases, including alcohol-associated liver disease, fibrosis, and HCC (see^4,5^ for recent reviews).

*PNPLA3* rs738409 c.444C<G encodes the PNPLA3-I148M mutant protein, which displays a reduced enzymatic function and higher stability. Evidence derived from mouse studies indicates that the accumulation of catalytically inactive PNPLA3 proteins at the surface of lipid droplets is associated with triglyceride accumulation. Whereas it is known that the variant protein PNPLA3-I148M contributes to liver damage and increases the damage by concomitant liver injury (eg, alcohol consumption, viral infection), the overall molecular mechanisms by which *PNPLA3* ^*I148M*^ favors fibrosis and carcinogenesis have yet to be fully defined. Bruschi et al have recently shown that primary human HSC harboring this polymorphism secrete higher levels of pro-inflammatory factors, like IL-8, RANTES, monocyte chemoattractant protein-1 (MCP-1), and growth-regulated oncogene (GROα), compared to cells expressing only PNPLA3-WT,^6^ indicating that this polymorphism may impact inflammation, in addition to its metabolic effects.

HCC typically arises in the background of chronic inflammation or cirrhosis and is characterized by the presence of cytokines originating from a broad range of cells, including HSC, immune and endothelial cells, and fibroblasts.^1^ Inflammatory cytokines, as well as growth factors, are involved in hepatocarcinogenesis, and several studies have revealed increased concentrations in serum or plasma samples of patients with chronic liver diseases including NAFLD and HCC (eg,^7–9^). Although the *PNPLA3* ^*I148M*^ variant has been extensively studied and is strongly associated with liver diseases, only few studies have been conducted to characterize potential changes in the serum cytokine profiles in individuals harboring this allele. To further study these potential associations, we measured the concentrations of 22 selected cytokines, chemokines, and growth factors in sera of 11 healthy donors, 91 patients with NAFLD (including 57 with simple steatosis, 10 with NASH, and 24 with cirrhosis), and 21 patients with HCC, who were all genotyped for this polymorphism. The results were used to assess the suitability of the analytes as diagnostic biomarkers in prediction models for steatosis, advanced NAFLD with complications, cirrhosis, or HCC.

## METHODS

### Donors

Sera from patients with NAFLD, either with “simple” steatosis or with NASH, with or without cirrhosis, and from patients with HCC were collected at the Department of Medicine II, Saarland University Hospital; samples from healthy individuals were obtained at the Institute for Occupational and Environmental Medicine and Public Health (IAUP), Saarland University (Homburg, Germany). In healthy controls, liver-specific diseases were excluded, and none presented with elevated liver function tests. Table 1 summarizes their characteristics. The studies were performed in agreement with the Declaration of Helsinki and national regulations. The Ethics Committees in Germany (Ethik-Kommission der Ärztekammer des Saarlandes, 271/11 - 79/12) and in Luxembourg (Comité National d’Éthique de Recherche, 201309/07) approved the study procedures. Written informed consent was obtained from all patients before inclusion in the study. After collection, whole blood samples were kept at room temperature for clotting. After 30 min, tubes were centrifuged at 2000 g for 10 min at 4°C. Serum samples were kept at −80°C until cytokine measurements. Genomic DNA was isolated from EDTA-anticoagulated blood samples using the membrane-based QIAamp DNA extraction protocol (Qiagen, Hilden, Germany).

**TABLE 1:** Characteristics of patients included in the cohort (n = 123)

	Healthy (N = 11)	NAFLD-NC-Stea (N = 57)	NAFLD-NC-NASH (N = 10)	NAFLD-Cirr (N = 24)	HCC-NC (N = 6)	HCC-Cirr (N = 15)	Overall (N = 123)
PNPLA3.I148M.variant, n (%)							
CC	6 (54.5)	28 (49.1)	6 (60.0)	11 (45.8)	2 (33.3)	2 (13.3)	55 (44.7)
CG	3 (27.3)	23 (40.4)	1 (10.0)	11 (45.8)	3 (50.0)	6 (40.0)	47 (38.2)
GG	2 (18.2)	6 (10.5)	3 (30.0)	2 (8.3)	1 (16.7)	7 (46.7)	21 (17.1)
Sex, n (%)							
F	6 (54.5)	27 (47.4)	7 (70.0)	12 (50.0)	1 (16.7)	4 (26.7)	57 (46.3)
M	5 (45.5)	30 (52.6)	3 (30.0)	12 (50.0)	5 (83.3)	11 (73.3)	66 (53.7)
Age							
Mean (SD)	52.6 (6.55)	56.9 (11.6)	48.1 (11.6)	60.4 (14.7)	66.3 (7.00)	65.1 (10.8)	57.9 (12.4)
Median [Min, Max]	52.0 [44.0, 67.0]	57.0 [23.0, 80.0]	47.5 [31.0, 69.0]	62.0 [31.0, 84.0]	64.5 [58.0, 77.0]	64.0 [45.0, 81.0]	58.0 [23.0, 84.0]
BMI							
Mean (SD)	29.8 (3.84)	30.9 (5.90)	30.5 (6.77)	25.9 (5.84)	30.8 (4.01)	29.8 (6.11)	29.7 (5.83)
Median [Min, Max]	28.2 [25.6, 38.3]	29.7 [17.6, 44.8]	31.6 [21.8, 41.0]	25.6 [17.3, 37.3]	30.2 [27.0, 38.1]	29.7 [22.9, 44.5]	29.1 [17.3, 44.8]
Missing	0 (0)	15 (26.3)	4 (40.0)	7 (29.2)	0 (0)	0 (0)	26 (21.1)

*Notes:* Demographics, *PNPLA3* genotype, and BMI are indicated for the 6 groups: healthy, NAFLD-NC-Stea, NAFLD-NC-NASH, NAFLD-Cirr, HCC-NC, HCC-Cirr. Age and BMI are presented as mean + SD and median. Numbers of missing values are also indicated.

Abbreviations: PNPLA3, patatin-like phospholipase domain-containing protein 3.

### Measurement of serum analytes

The serum concentrations of cytokines, chemokines, growth factors, and soluble receptors were measured using the Bio-Plex 200 (Bio-Rad) device (we often use the global term “cytokines” to refer to all these analytes). Pilot experiments were made for a total of 59 cytokines. Twenty-two cytokines were further studied with a customized kit (see Supplemental Digital Content for further information, http://links.lww.com/HC9/A633). Bio-Plex Pro Cytokine assays were performed according to the manufacturer’s instructions (Bio-Rad, Temse, Belgium). The data obtained were analyzed using the Bio-Plex Data Pro software version 1.02 (Bio-Rad).

### Genotyping of PNPLA3^I148M^ risk variant

The *PNPLA3* polymorphism rs738409 was genotyped using a PCR-based assay with 5’-nuclease and fluorescence detection (TaqMan^®^, Life Technologies, Darmstadt, Germany; assay number C_7241_10).

### Statistical analysis

Differences between groups (for the 22 cytokines or clinical variables) were tested using the nonparametric Dunn test. We only included clinical parameters in the statistical analyses if at least 10 values were available. When there were several groups, *p* values of pairwise comparisons were adjusted within each variable using the Holm method. If only 2 groups were tested, a Benjamini-Hochberg correction^10^ was applied between variables. Associations between categorical factors were performed using a chi-square test. Correlations between cytokines or numeric clinical parameters (with at least 10 pairs of values) were analyzed with Pearson correlation. Before those analyses, cytokine measurements and clinical parameters were first inspected regarding their distribution and log-transformed when necessary to better match normal distribution. Values for all cytokines were log-transformed, except for IL-17A, IL-4, and RANTES, as well as values for the clinical parameters C-reactive protein and transient elastography. The basis of the predictions was a generalized linear model using a binomial distribution. This model predicts the binary class of a dependent variable (y), given one or several independent variable(s). It was run for each of the 22 cytokines, in a transformed scale when necessary. When this generalized linear model showed a Benjamini-Hochberg adjusted *p*-value < 0.05, we selected it for further ROC analysis, using the initial class as the “truth”. The cutoff represents the threshold in y value (class prediction). In the case of Benjamini-Hochberg adjusted *p*-value < 0.05, we also built models with more variables (second cytokine or clinical parameter or genotype), with the maximum of 1 predictor per 10 events (“rule-of-thumb limit”). When several variables were included in a model, we excluded correlated pairs of variables (ie, *p* > 0.05, R > 0.4, or R <− 0.4) to avoid collinearity. All data analysis was done in R version 4.2.2.^11^ Graphs were obtained using ggplot2^12^ and corrplot.^13^ ROC curves were obtained using ROCit package.^14^

## RESULTS

### The study cohort: 123 individuals, all genotyped for PNPLA3

We measured cytokine concentrations in sera of patients with different stages of NAFLD and liver tumors, comprising 91 individuals with NAFLD (24 with cirrhosis, “NAFLD-Cirr”; 57 with “simple” steatosis, “NAFLD-non-cirrhosis (NC)-Stea”; and 10 with NASH but without cirrhosis, “NAFLD-NC-NASH”) and 21 individuals with HCC (15 with cirrhosis, “HCC-Cirr”, and 6 without, “HCC-NC”). The control cohort comprised 11 healthy persons without chronic liver diseases (“controls”). The demographics and clinical characteristics of cases and controls are presented in Table 1. All persons enrolled were genotyped for their allele status of *PNPLA3*. Overall, 44.7% of the cohort are homozygous for the WT allele (CC), 38.2% are heterozygous (CG), and 17.1% are homozygous for the risk allele (GG).^15^

### Altered cytokine profiles in sera from patients with chronic liver diseases

**FIGURE 1:**
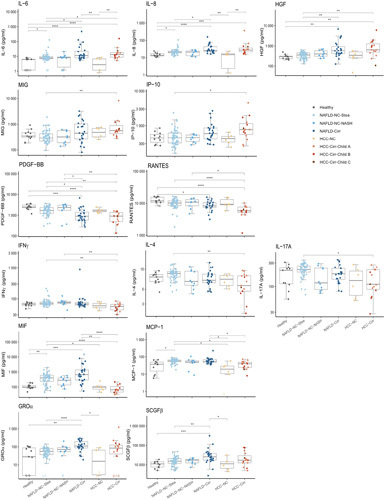
Profiles of selected serum cytokines in patients with chronic liver diseases. Values used for the graphical representation were log-transformed. The horizontal line within the box plot represents the median, and vertical lines from the boxes (whiskers) indicate the variability outside the upper and lower quartiles. Empty circles represent values “out of range” (see supplements for further information). Statistical analysis was done on non-logged data; Kruskal-Wallis H and post hoc Dunn’s multiple comparison tests were performed. *p* values of pairwise comparisons were adjusted for each cytokine using the Holm method. ****p < 0.0001, ***p < 0.001, **p < 0.01, *p < 0.05. Abbreviations: MCP-1, monocyte chemoattractant protein-1; MIF, macrophage migration inhibitory factor.

We assessed the serum cytokine levels by multiplex ELISA assays. Twenty-two cytokines were included in the further analysis. Table 2 presents their median values and ranges; Figure 1 depicts results for the 14 most relevant analytes.

**TABLE 2:** Levels of cytokines in the sera of patients and of healthy controls

	Healthy (n = 11)	NAFLD-NC-Stea (n = 57)	NAFLD-NC-NASH (n = 10)	NAFLD-Cirr (n = 24)	HCC-NC (n = 6)	HCC-Cirr (n = 15)
Cytokine	Median range	Median range	Median range	Median range	Median range	Median range
βNGF	1.23 (0.62–1.23)	1.14 (0.62–39.99)	1.18 (0.62–17.65)	6.11 (0.76–32.73)^a^	0.92 (0.62–6.29)	1.23 (1.14–38.33)
CTACK	463.23 (184.42–818.44)	254.14 (3.2–838.92)	341.69 (105.27–920.24)	368.73 (69.62–1088.71)	463.77 (143.6–638.7)	301.68 (55.23–1299.55)
**GROα**	28.04 (2.54–109.22)	55.69 (2.44–141.03)	72.3 (2.54–204.9)	110.32 (19.31–296.99)	34.55 (2.54–89.07)	84.46 (2.44–1211.7)
**HGF**	289.71 (200.05–490.23)	350.78 (129.56–642.61)	397.02 (235.02–701.35)	575.93 (125.97–6830.61)	341.88 (47.91–574.89)	646.6 (106.79–6043.4)
IFNα2	21.64 (1.82–28.05)	19.14 (2.4–129.94)	16.11 (1.82–63.26)	26.6 (2.4–300.79)	21.29 (1.82–24.5)	28.85 (12.77–81.07)^b^
**IFNγ**	67.22 (47.85–79.33)	70.91 (53.57–213.25)	75.5 (43.16–123.76)	68.31 (55.03–882.89)	60.16 (41–81.19)	56.88 (30.65–84.57)
IL-16	103.58 (36.58–174.93)	105.87 (1.74–330.03)	95.46 (50.68–579.47)	128.76 (74.5–2361.91)	87.53 (13.92–143.59)	123.1 (74.37–387.49)
**IL-17A**	212.45 (55.72–310.21)	220.9 (68.56–343.26)	118.47 (73.39–291.56)	180.6 (79.86–348.68)	136.8 (52.52–278.47)	111.12 (27.85–274.45)
IL-1Ra	72.75 (48.27–144)	92.15 (46.73–505.91)	105.85 (70.43–380.78)	84.78 (59.62–2246.1)	74.79 (40.83–136.1)	67.32 (29.47–140.09)^c^
**IL-4**	7.62 (4.52–9.45)	8.61 (4.58–12.06)	6.54 (4.26–10.9)	6.8 (4.53–12.6)	7.1 (5.04–8.47)	5.66 (2.15–9.55)
**IL-6**	6.09 (1.17–8.04)	7.67 (1.17–129.08)	8 (1.17–20.49)	11.09 (5.31–478.13)	3.77 (0.78–8.32)	13.11 (5.2–159.62)
**IL-8**	13.48 (10.42–24.14)	21.13 (11.17–106.31)	20.9 (11.02–28.48)	25.85 (16–158.23)	14.72 (1.29–23.59)	27.67 (15.22–368.12)
IL-9	53.01 (38.28–74.07)	50.11 (24.27–139.36)	56.38 (13.03–78.63)	38.67 (17.15–804.29)	49.37 (34.6–58.8)	30.53 (13.77–53.33)^a^ ^b^
**IP-10**	442.96 (264.82–853.38)	448.58 (160.42–2351.68)	454.6 (278.65–582.88)	593.09 (281.56–2669.84)	422.94 (239.2–649.62)	774.68 (259.08–4548.49)
**MCP-1**	37.36 (5.91–67.88)	58.25 (18.12–92.3)	50.62 (8.86–105.85)	54.39 (35.05–231.87)	20.47 (0.74–57.39)	39.88 (8.13–72.62)
M-CSF	0.84 (0.56–6.86)	0.51 (0.51–21.59)	0.62 (0.51–4.63)	0.76 (0.51–51.89)	0.62 (0.56–6.75)	5.49 (0.56–23.55)^d^
**MIF**	100.2 (47.4–192.15)	402.9 (43.62–1958.45)	283.32 (74.21–567.47)	667.36 (53.73–14982.36)	96.13 (44.81–252.63)	65.21 (32.37–216.4)
**MIG**	347.1 (184.71–924.94)	291.72 (95.13–1898.94)	322.84 (137.3–622.71)	449.18 (80.13–3225.42)	507.78 (251.58–760.46)	566.75 (278.16–8252.32)
**PDGF-BB**	2509.38 (1183.83–3644.99)	1700.61 (301.89–3723.67)	2381.78 (304.75–3412.07)	906.14 (271.03–6821.36)	1661.12 (1273.21–2463.38)	870.98 (130.27–1523.28)
**RANTES**	11807.52 (8319.52–16244.62)	10230.69 (1814.6–16373.41)	10829.96 (4733.63–14394.48)	8275.75 (3163.89–15414.77)	9303.26 (5856.75–15241.56)	5842.51 (1169.86–10180)
**SCGFβ**	11135.86 (5105.29–18790.75)	15256.66 (5039.05–47683.67)	17219.36 (7390.16–22538.39)	25821.33 (2739.75–310786.8)	11516.61 (4484.61–31404.77)	17231.88 (5001.24–78089.52)
TRAIL	16.58 (0.93–67.21)	21.57 (0.42–1688.2)	26.01 (0.42–685.87)	18.66 (0.42–1639.94)	25.1 (0.93–60.86)	11.66 (0.67–129.4)

*Notes:* Concentrations are indicated in pg/mL, and median values and ranges are represented. Analytes printed in bold are presented graphically in Figure 1. For the remaining 8 cytokines, any significant differences are indicated.

Statistical analysis was done on non-logged data; Kruskal-Wallis H and post hoc Dunn’s multiple comparison tests were performed. *p* values of pairwise comparisons were adjusted for each cytokine using the Holm method.

a compared to healthy controls.

b to NAFLD-NC-Stea.

c to NAFLD-NC-NASH.

d *p* < 0.0001, a, b, c: *p* < 0.05.

Abbreviations: CTACK, cutaneous T cell-attracting chemokine; GROα: growth-regulated oncogene; MCP-1, monocyte chemoattractant protein-1; M-CSF, macrophage colony-stimulating factor; MIF, macrophage migration inhibitory factor; MIG, monokine induced gamma; NGF, nerve growth factor; RANTES, regulated on activation, normal T cell expressed and secreted; SCGF: stem cell growth factor.

### Increased levels of IL-6, HGF, and IL-8 in cirrhosis, decreased levels of platelet derived growth factor-BB (PDGF-BB) and RANTES

The serum concentrations of the inflammatory cytokines IL-6 and IL-8 and of the growth factor HGF were increased in patients with NAFLD and HCC compared to controls, the increase being particularly evident in patients with cirrhosis (median values for IL-6: 6.1, 11.1, 13.1 pg/mL for control, NAFLD-Cirr, and HCC-Cirr samples, respectively; IL-8: 13.5, 25.9, and 27.7 pg/mL; HGF: 290, 576, and 647 pg/mL, Figure 1 and Table 2). Patients with simple steatosis had significantly higher levels of IL-6 and IL-8 compared to controls. Serum concentrations of the chemokines monokine induced gamma (MIG) and IP-10 had the highest levels in patients with HCC with cirrhosis, reaching statistical significance in comparison to the steatosis group.

On the other hand, serum levels of the growth factor PDGF-BB and of the chemokine RANTES were reduced compared to the healthy controls, with the lowest levels detected in patients with cirrhosis. Interferon (IFNγ), IL-4, and IL-17 concentrations were the lowest in patients with HCC with cirrhosis. Their concentration differences were statistically significant in comparison to patients with simple steatosis (IL-4 and IL-17) or to both groups of noncirrhosis NAFLD (IFNγ) (Figure 1).

### MIF and MCP-1 are elevated in sera of patients with NAFLD

Interestingly, the chemokines macrophage migration inhibitory factor (MIF) and MCP-1 were found at higher concentrations in sera of patients with NAFLD compared to healthy donors and patients with HCC (Figure 1 and Table 2). The concentrations of stem cell growth factor (SCGFβ) and GROα were significantly increased in patients with NAFLD with cirrhosis compared to patients with NAFLD with simple steatosis and healthy controls.

### Correlation analyses

We analyzed whether significant correlations can be observed between pairs of cytokines.

A graphical overview of the pairwise correlations for all 22 cytokines as well as plots for those pairs with *R*-values ≥ 0.6 is provided in Supplemental Figures S1A and B, http://links.lww.com/HC9/A629. IL-17 and IL-4 have the highest correlation with an (overall) *R*-value of 0.85. Further analyses reveal that this correlation is highly significant also in 4 different subgroups: healthy: *R* = 0.93; NAFLD-NC-Stea: *R* = 0.79; NAFLD-Cirr: *R* = 0.79; HCC-Cirr: *R* = 0.93 (Supplemental Figure S1C, http://links.lww.com/HC9/A629, also depicting other pairs showing a high correlation in more than 1 subgroup). Interestingly, while IL-1Ra has a negative correlation with MCP-1 in healthy samples (*R*=− 0.76), it shows a positive correlation (*R* = 0.8) in NAFLD-Cirr samples (Supplemental Figure S1C, http://links.lww.com/HC9/A629).

We also assessed possible correlations between cytokine concentrations and clinical parameters. Not surprisingly, we observed high correlations between levels of the inflammation marker C-reactive protein and IL-6 (*R* = 0.67), IL-8 (*R* = 0.70), and IL-16 (*R* = 0.79) in sera of patients with HCC (n = 20, adj. *p*-value < 0.05), as depicted in Supplemental Figure S2A, http://links.lww.com/HC9/A630. Interestingly, the results of the measurements of liver stiffness (transient elastography performed with “FibroScan”), available for the patients with NAFLD and healthy controls (98 samples in total), correlate well with the concentrations of circulating HGF (*R* = 0.64, Supplemental Figure S2B, http://links.lww.com/HC9/A630). The corresponding *R*-values for correlations between elastography and IL-8 or SCGFβ are lower (0.47, 0.44) but still statistically significant (adj. *p*-value < 0.001).

To avoid collinearity in subsequent prediction analyses, combinations of 2 parameters were excluded if they had an overall *R*-value > 0.4.

### Cytokines as predictive factors for liver diseases

Using ROC curve analysis, we analyzed the discriminative power of the cytokines for the prediction of steatosis, cirrhosis, or HCC.

### MIF, IL-8, and IL-6 have the highest predictive value for steatosis detection

**FIGURE 2:**
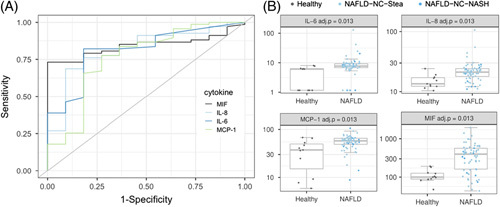
ROC curve analysis for prediction of noncirrhotic NAFLD versus healthy cases. (A): ROC curves for the 4 cytokines with p. adj. < 0.05: MIF, IL-8, IL-6, MCP-1. (B): Concentration plots for MIF, IL-8, IL-6, and MCP-1 for the healthy group (11 samples) and the noncirrhotic NAFLD group (57 NAFLD-NC-Stea + 10 NAFLD-NC-NASH samples). Abbreviations: MCP-1, monocyte chemoattractant protein-1; MIF, macrophage migration inhibitory factor.

We first searched among the measured cytokines for predictors of NAFLD, focusing on patients without cirrhosis and without HCC. To this end, the data on serum cytokine concentrations obtained from patients with NAFLD without cirrhosis (NAFLD-NC-Stea, NAFLD-NC-NASH) and from healthy controls were included in the analysis. For 4 of the cytokines, MIF, IL-8, IL-6, and MCP-1, the analysis yielded adj. *p* values <0.05 (Figure 2A/B). MIF, IL-8, and IL-6 have AUC values > 0.8, high PPVs, and high sensitivities. However, their specificities and NPVs are low (Table 3).

**TABLE 3:** ROC analyses

cytokine (x) OR cytokines (x1_x2)	AUC	PPV	NPV	SENS	SPEC	Cutoff	Equation
Prediction: Steatosis vs. healthy, 1 cytokine (Figure 2A)							
MIF	0.84	0.86	NaN	1	0.00	0.36	y ∼ –7.07+3.95 × x
IL-8	0.82	0.87	1	1	0.09	0.46	y ∼ –10.7+10.10 × x
IL-6	0.81	0.86	NaN	1	0.00	0.51	y ∼ –0.14+2.80 × x
MCP-1	0.76	0.88	1	1	0.18	0.32	y ∼ –4.4+3.84 × x
Prediction: Cirrhosis within NAFLD, 2 cytokines (Figure 3A)							
HGF_PDGF–BB	0.86	0.94	0.89	0.67	0.99	0.54	y ∼ –6.12+6.15 × x1+–3.61 × x2
GROα_PDGF–BB	0.85	0.64	0.95	0.88	0.82	0.24	y ∼ –1.88+5.95 × x1+–3.31 × x2
M–CSF_PDGF–BB	0.84	0.61	0.93	0.83	0.81	0.24	y ∼ 8.35+1.45 × x1+–3.06 × x2
GROα_MIG	0.83	0.77	0.90	0.71	0.93	0.37	y ∼ –13+4.76 × x1+1.17 × x2
βNGF_PDGF–BB	0.83	0.69	0.91	0.75	0.88	0.33	y ∼ 6.8+1.32 × x1+–2.70 × x2
IL-6_PDGF–BB	0.83	0.65	0.89	0.71	0.87	0.32	y ∼ 5.05+2.70 × x1+–2.85 × x2
PDGF–BB_SCGFβ	0.83	0.80	0.89	0.67	0.94	0.46	y ∼ –10.2+–2.97 × x1+4.29 × x2
GROα_HGF	0.83	1.00	0.87	0.58	1.00	0.54	y ∼ –18.4+3.81 × x1+3.89 × x2
GROα_IP–10	0.82	0.73	0.88	0.67	0.91	0.40	y ∼ –14.61+4.69 × x1+1.74 × x2
CTACK_GROα	0.82	0.82	0.86	0.58	0.96	0.47	y ∼ –14.61+4.69 × x1+1.74 × x2
Prediction: NAFLD complication, 1 or 2 cytokines (Figure 3C)							
GROα_IL–17A	0.82	0.69	0.85	0.77	0.79	0.40	y ∼ –4.73+3.84 × x1+–0.01 × x2
GROα_IL–4	0.81	0.68	0.85	0.77	0.77	0.36	y ∼ –3.46+3.37 × x1+–0.40 × x2
CTACK_GROα	0.79	0.75	0.82	0.69	0.86	0.47	y ∼ –10.39+1.95 × x1+2.81 × x2
HGF_IL–4	0.78	0.73	0.85	0.77	0.82	0.39	y ∼ –6.78+3.59 × x1+–0.39 × x2
HGF_IL–17A	0.78	0.67	0.83	0.74	0.77	0.39	y ∼ –8.04+3.71 × x1+–0.01 × x2
GROα	0.78	0.77	0.81	0.66	0.88	0.47	y ∼ –6.24+3.14 × x
GROα_HGF	0.77	0.75	0.82	0.69	0.86	0.43	y ∼ –11.43+2.37 × x1+2.55 × x2
CTACK_IL–4	0.76	0.52	0.85	0.86	0.51	0.29	y ∼ –3.4+2.41 × x1+–0.38 × x2
CTACK_IL–17A	0.75	0.66	0.79	0.66	0.79	0.42	y ∼ –3.96+2.14 × x1+–0.01 × x2
CTACK_HGF	0.75	0.52	0.83	0.83	0.53	0.29	y ∼ –13.14+1.81 × x1+3.15 × x2
Prediction: HCC vs. NAFLD, 1 cytokine (Figure 4A, Figure 5C)							
MIF	0.91	0.57	0.94	0.76	0.87	0.30	y ∼ 9.17+–4.86 × x
MCP-1	0.81	0.69	0.90	0.52	0.95	0.33	y ∼ 6.9+–5.14 × x
IFNγ	0.77	0.91	0.89	0.48	0.99	0.45	y ∼ 10.03+–6.01 × x
M-CSF	0.75	0.32	1.00	1.00	0.52	0.12	y ∼ 2.04+–0.49 × x
RANTES	0.75	0.58	0.89	0.52	0.91	0.30	y ∼ 0.57+–0.01 × x
IL-4	0.73	0.50	0.88	0.48	0.89	0.33	y ∼ 1.05+–0.00 × x
MIG	0.71	0.33	0.90	0.67	0.69	0.19	y ∼ –1.71+1.05 × x
IL-17A	0.71	0.55	0.89	0.52	0.90	0.33	y ∼ –6.83+2.01 × x
IL-1Ra	0.69	0.75	0.88	0.43	0.97	0.38	y ∼ 4.71+–2.02 × x
PDGF-BB	0.68	0.30	0.94	0.86	0.54	0.15	y ∼ 10.03+–6.01 × x
IL-9	0.66	0.26	0.97	0.95	0.37	0.14	y ∼ 3.39+–3.03 × x
Prediction: HCC vs. NAFLD, 2 cytokines (Figure 4C)							
MIF_MIG	0.95	0.77	0.96	0.81	0.95	0.44	y ∼ –4.04+–6.84 × x1+6.42 × x2
IL–4_MIF	0.94	0.84	0.95	0.76	0.97	0.46	y ∼ 18.34+–0.80 × x1+–6.62 × x2
IL–17A_MIF	0.94	0.64	0.96	0.86	0.89	0.40	y ∼ 14.97+–0.02 × x1+–6.39 × x2
MIF_PDGF–BB	0.94	0.68	0.95	0.81	0.91	0.31	y ∼ 21.96+– 5.61 × x1 +– 3.63 × x2
M–CSF_MIF	0.93	0.65	0.95	0.81	0.90	0.32	y ∼ 9.6+1.88 × x1+– 5.27 × x2
MIF_RANTES	0.93	0.77	0.96	0.81	0.95	0.42	y ∼ 13.61+– 5.31 × x1+– 0.00039 × x2
MCP–1_MIF	0.93	0.59	0.98	0.90	0.86	0.29	y ∼ 14.53+– 3.50 × x1+– 4.83 × x2
IL–9_MIF	0.92	0.71	0.93	0.71	0.93	0.42	y ∼ 17.1+– 4.49 × x1+– 5.21 × x2
Prediction: HCC vs. NAFLD, cytokine & PNPLA3 (Figure 5C)							
MIF	0.92	0.68	0.93	0.71	0.92	0.40	y ∼ 8.26+1.13 × *PNPLA3*variant+– 4.78 × x
MCP-1	0.85	0.47	0.92	0.71	0.81	0.20	y ∼ 6.24+1.69 × *PNPLA3*variant+– 5.46 × x
IFNγ	0.82	1.00	0.88	0.43	1.00	0.55	y ∼ 22.93+1.44 × *PNPLA3*variant+– 14.03 × x
IL-4	0.78	0.40	0.94	0.81	0.71	0.78	y ∼ 1.04+1.41 × *PNPLA3*variant+–0.48 × x
IL-17A	0.78	0.36	0.94	0.81	0.67	0.16	y ∼ –0.4+1.46 × *PNPLA3*variant+– 0.01 × x
RANTES	0.78	0.65	0.89	0.52	0.93	0.39	y ∼ 0.06+1.30 × *PNPLA3*variant+– 0.00028 × x
M-CSF	0.77	0.40	0.94	0.81	0.73	0.18	y ∼ –2.53+1.27 × *PNPLA3*variant+0.93 × x
PDGF-BB	0.77	0.44	0.95	0.81	0.76	0.22	y ∼ 3.94+1.45 × *PNPLA3*variant+– 2.09 × x
IL-1Ra	0.77	0.52	0.91	0.62	0.80	0.28	y ∼ 8.69+1.32 × *PNPLA3*variant+– 5.76 × x
MIG	0.76	0.41	0.91	0.67	0.78	0.22	y ∼ –7.33+1.33 × *PNPLA3*variant+1.87 × x
IL-9	0.74	0.36	0.94	0.81	0.67	0.20	y ∼ 2.42+1.43 × *PNPLA3*variant+– 3.02 × x

*Notes:* For the ROC analyses shown in the indicated figures, the AUC values, PPV, NPV, and the values for sensitivity (SENS) and specificity (SPEC) are provided. The cutoffs refer to the outcome of the regression (y) (not to absolute cytokine concentrations). NaN: not a number; x: cytokine (single); x1, x2: cytokines (for combinations); y: prediction model. Note that the values x, x1, and x2 refer to log-transformed cytokine concentrations, with the following exceptions: IL-17A, IL-4, and RANTES.

Abbreviations: CTACK, cutaneous T cell-attracting chemokine; GROα: growth-regulated oncogene; MCP-1, monocyte chemoattractant protein-1; M-CSF, macrophage colony-stimulating factor; MIF, macrophage migration inhibitory factor; MIG, monokine induced gamma; NGF, nerve growth factor; PDGF-BB, platelet derived growth factor-BB; *PNPLA3*, *patatin-like phospholipase domain-containing protein 3;* RANTES, regulated on activation, normal T cell expressed and secreted; SCGF, stem cell growth factor.

### HGF and PDGF-BB are the best predictive factors for cirrhosis

As shown in Figure 1, profiles for several cytokines seem to be more “pronounced” in patients with cirrhosis, compared to the corresponding patients with NAFLD or HCC without cirrhosis. We, therefore, performed ROC analyses to identify factors that best discriminate between the presence and absence of cirrhosis. When including the data for the entire cohort, PDGF-BB and HGF yielded the highest AUC values of 0.81 and 0.80, respectively, followed by IL-6 and IL-8 (0.78), RANTES (0.77), and 11 further cytokines yielding adj. *p* values < 0.05 (Supplemental Figure S3A/B, http://links.lww.com/HC9/A631).

To further improve the model, another cytokine was added as a second parameter, excluding any combinations of cytokines correlating with each other (threshold: IRI = 0.4, see Supplemental Figure S1A, http://links.lww.com/HC9/A629). The pair HGF/PDGF-BB achieved the highest AUC score of 0.88 (Supplemental Figure S3C/D, http://links.lww.com/HC9/A631). This top AUC value was also found for the combinations IL-17A/IL-6 and M-CSF/PDGF-BB, while combinations of PDGF-BB with IL-8, IL-6, or βNGF resulted in slightly lower AUC values (0.87), still with high scores (≥0.7) for PPV, NPV, sensitivity, and specificity (Supplemental Figure S3C/D, http://links.lww.com/HC9/A631).

**FIGURE 3:**
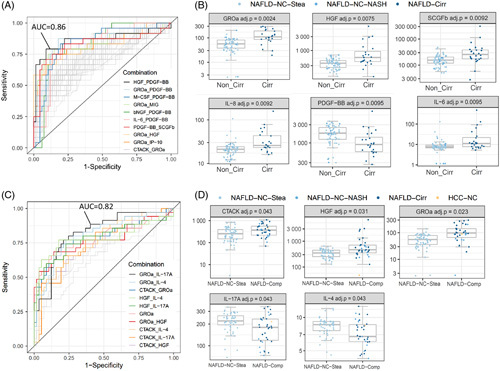
ROC curve analysis for prediction of cirrhosis or “complication” within the NAFLD group. (A): ROC curves for the 10 cytokine pairs discriminating best between cirrhosis and noncirrhosis are highlighted in colors. (B): Concentration plots for the top 6 cytokines (see Supplemental Figure S4A, http://links.lww.com/HC9/A632), the noncirrhotic group comprising 67 samples (NAFLD-NC-Stea as well as NAFLD-NC-NASH), the cirrhotic group comprising 2 samples. (C): ROC curves for the 10 cytokine pairs discriminating best between “complicated” (NAFLD-NC-NASH, NAFLD-Cirr, and NAFLD-related HCC, 35 samples) and “uncomplicated” (NAFLD-NC-Stea, 57 samples) are highlighted in colors. (D): Concentration plots for the top 5 cytokines (see Supplemental Figure S4C, http://links.lww.com/HC9/A632).

When focusing the cirrhosis prediction on the NAFLD group, GROα and HGF were the best single predictors with AUC values of 0.82 and 0.77, respectively, among 13 cytokines yielding results with adj. *p* values < 0.05 (Supplemental Figure S4, http://links.lww.com/HC9/A632). When a second cytokine was added to the analysis (Figure 3A), the combination HGF/PDGF-BB showed the highest AUC value (0.86), as previously observed for the entire cohort (Supplemental Figure S3C/D, http://links.lww.com/HC9/A631). The cytokine pair GROα/PDGF-BB scored second (AUC: 0.85), whereas the combination M-CSF/PDGF-BB yielded an AUC value of 0.84 (Table 3).

An additional analysis was performed to discriminate NAFLD without complications (corresponds to the NAFLD-NC-Stea samples) from advanced NAFLD with complications, the latter group comprising patients with NASH, NAFLD-associated cirrhosis, or NAFLD-related HCC. As shown in Figure 3C/D and in Table 3, GROα performed best as a single predictor (AUC: 0.78). This value was enhanced upon adding IL-17, IL-4, or CTACK as second parameter (AUC values 0.82, 0.81, and 0.79, respectively).

### The combination of MIF & MIG performs best to discriminate HCC from non-HCC among patients with chronic liver diseases (AUC: 0.95)

**FIGURE 4:**
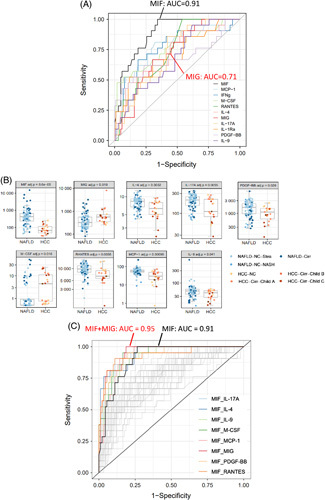
ROC curve analysis for prediction of HCC versus non-HCC among patients with chronic liver diseases. (A): ROC curves for 11 cytokines, allowing best to distinguish HCC versus non-HCC. (B): Concentration plots for cytokines with an adjusted *p*-value > 0.05 are shown for the non-HCC group (91 NAFLD samples) and the HCC group (21 samples) (C): Cytokine pairs are depicted by colored lines if their AUC is higher than the AUC of MIF alone. Abbreviations: MCP-1, monocyte chemoattractant protein-1; MIF, migration inhibitory factor.

Finally, we performed ROC analyses for HCC versus “non-HCC” samples, the latter including all the NAFLD samples but not the healthy controls. As shown in Figure 4A–C and in Table 3, among the 11 cytokines with adj. *p* values < 0.05, MIF and MCP-1 performed best. MIF shows the highest AUC (0.91), high scores for NPV (0.94), and specificity (0.87) but a lower PPV (0.57) and a sensitivity score of 0.76. MCP-1 has an AUC of 0.81, a PPV of 0.69, and an NPV of 0.90. While its value for specificity is very high (0.95), the sensitivity score is low (0.52).

The combination of MIF with MCP-1 slightly improved the model compared to MIF alone (0.93 vs. 0.91); the same value could be achieved by combination with M-CSF or RANTES. Whereas the combination of MIF with IL-4, IL-17A, or PDGF-BB led to AUC values of 0.94 (Figure 4C, Table 3), the association of MIF with MIG as second parameter increased the AUC from 0.91 to 0.95, leading to the best predictive model for discrimination of HCC within this cohort of individuals with chronic liver diseases. Next to a high score for sensitivity (0.81) and a PPV of 0.77, this combination has excellent values for specificity (0.95) and an NPV of 0.96. Other pairs yielding high AUC values are combinations of MCP-1 with RANTES, M-CSF, MIG, or PDGF-BB (0.89, 0.87, 0.86, and 0.84, respectively) and combinations of IFNγ with M-CSF (0.83), MIG, or MCP-1 (0.82) (data not shown).

### The PNPLA3^I148M^ variant does not have significant effects on serum cytokine patterns

**FIGURE 5:**
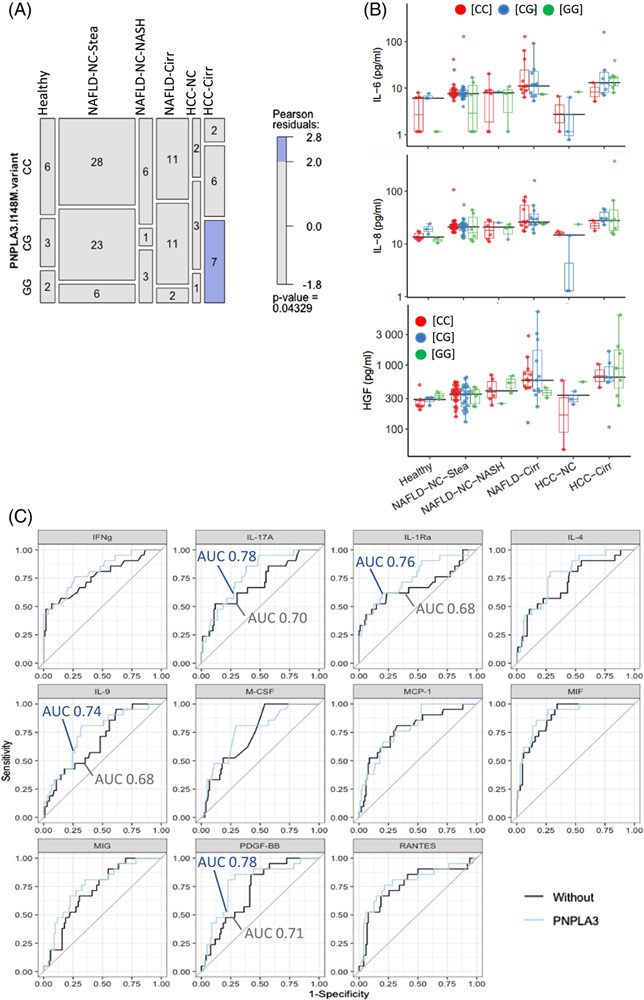
The *PNPLA3* c.444C > G, rs738409 risk allele overrepresented in patients with HCC does not strongly affect levels of circulating cytokine but slightly improves HCC prediction models based on single cytokines. (A): Graphical representation of the patient cohort, indicating numbers of patients in the different disease groups and of healthy controls as well as their *PNPLA3* allele states [(CC), (CG), (GG), the G allele encoding the PNPLA3.I148M variant]. (B): No statistical difference regarding the concentrations of circulating cytokines was detected for the different allele states, as shown here for IL-6, IL-8, and HGF. (C): For the 11 cytokines yielding adj. *p* values < 0.05 for HCC prediction versus non-HCC (see Figure 4A), ROC curves with the *PNPLA3* allele status as the second parameter are shown. The AUC values are indicated when they increased by ≥ 0.06 upon inclusion of the *PNPLA3* genotype. Abbreviations: PNPLA3, *patatin-like phospholipase domain-containing protein 3*.

All persons enrolled in the cohort were genotyped for the common *PNPLA3* variant (Table 1). As reported,^15^ the *PNPLA3* risk allele c.444C>G, rs738409 encoding the PNPLA3-I148M variant is overrepresented in patients with HCC, where the [CG] and [GG] allele combinations are present in more than 80% of the patients (Figure 5A).

We assessed whether levels of serum cytokines within the pathology groups might be affected by the *PNPLA3* allele status. In general, no or only minor differences could be detected between the groups [CC], [CG], and [GG], but analyses with our small study cohort are certainly statistically underpowered to detect more subtle differences between groups. Of note, the highest serum concentrations of HGF, IL-6, and IL-8 were observed in carriers of the *PNPLA3* risk genotypes [CG] or [GG] (Figure 5B).

Interestingly, the addition of the *PNPLA3* genotype as second parameter to the models built with single cytokines improved the predictive power to discriminate HCC from the NAFLD samples. The highest AUC values were obtained with the combinations of *PNPLA3* with MIF (0.92 vs. 0.91 for the single cytokine model), MCP-1 (0.85 vs. 0.81), and IFNγ (0.82 vs. 0.77). Increases in the AUC value (by 0.07–0.09) could also be observed for IL-17A, IL-1Ra, PDGF-BB, and IL-9 upon inclusion of the *PNPLA3* genotype as second parameter (Figure 5C, Table 3). For the other prediction analyses, however, the addition of the *PNPLA3* genotype as a second or third parameter did not improve the discriminative power (data not shown).

## DISCUSSION

### Distinct patterns of circulating cytokines in chronic liver diseases and their diagnostic performance in prediction models

Our study confirms previous studies reporting that patients with chronic liver diseases display higher concentrations of circulating HGF, in particular those with cirrhosis. Higher HGF levels have been reported in sera of patients with HCC compared to healthy controls and could be linked to a higher Child score and shorter survival.^7,16^ In our cohort, we do not observe a statistically significant difference between patients with cirrhotic HCC and NAFLD (see Figure 1), in contrast to the results obtained by Pocino et al.^17^ HGF is part of a panel of 3 serum markers that predicts liver stiffness and fibrosis stages in patients with chronic liver diseases.^18^ In our present study, HGF also performs very well in models for cirrhosis prediction (Figure 3 **/**Supplemental Figure S3, http://links.lww.com/HC9/A631/Supplemental Figure S4, http://links.lww.com/HC9/A632). While HGF signaling through its receptor c-Met has been reported to have a beneficial role in preventing fibrosis and NASH,^19,20^ HGF/c-met signaling is considered pro-oncogenic for HCC where its inhibition is thus a therapeutic strategy (eg, clinical trial NCT02115373 with c-Met inhibitor Tepotinib).

In line with previous work, we found concentrations of IL-6 and IL-8 to be increased in patients with NAFLD and HCC, with the highest levels in the sera of patients with cirrhosis. High levels of these cytokines have been reported to be linked to a worse prognosis. For example, serum levels of both IL-6 and IL-8 have biomarker potential for the prediction of tumor response and overall survival in patients with hepatic malignancies undergoing transarterial chemoembolization.^21^ Interestingly, a recent proteo-transcriptomic study on NAFLD disease signatures identified IL-8 (=C-X-C motif ligand 8) to be present at a significantly higher level in the plasma proteomes of patients with fibrosis and NAS ≥ 4; a corresponding increase of IL-8 mRNA was found in hepatic cells with a restriction to macrophages.^3^ Work by the NASH clinical research network revealed that IL-8 and MCP-1 (but not IL-6) were among the factors that were significantly different between plasma samples of patients with NAFLD and severe fibrosis and those with no or mild fibrosis.^8^ However, Goyale et al proposed IL-6 as a prognostic factor for NAFLD progression as serum IL-6 was increased in patients with NAFLD with FibroScan values ≥ 7.2 kPa compared to those with lower scores for liver stiffness.^22^ We observed a slight correlation between IL-6 levels and FibroScan values (R=0.32, adj. *p*-value: 0.0116, data not shown).

In our cohort, the atypical chemokine-like factor MIF was present at higher concentrations in NAFLD sera, particularly in sera from patients with cirrhosis, whereas its concentration was lowest in sera of patients with HCC in cirrhosis. It has a high predictive value not only for the discrimination of (noncirrhotic) NAFLD versus healthy (Figure 2) but also for the discrimination of HCC versus non-HCC (NAFLD) (Figure 4, highest AUC for MIF and MIG: 0.95). MIF is a multifunctional factor present at a higher concentration in sera of obese persons and in individuals with type-2 diabetes, insulin resistance, and hepatic comorbidities.^23^ MIF expression was found to be increased in liver biopsies of patients with NAFLD with fibrosis compared to those with no or only mild fibrosis.^24,25^ Studies with MIF knock-out mice in models of chronic metabolic injury imply that MIF can exert both hepatoprotective as well as pro-fibrotic effects,^25,26^ indicating that the role of MIF in metabolic liver diseases may be complex and context-dependent. We did not find statistically significant differences in MIF levels between HCC and healthy samples (see Figure 1). Similarly, no significant differences in serum concentration between (primary and secondary) patients with liver cancer and healthy controls were observed in a recent study by Wirtz et al.^27^ They noted, however, that a lower level of MIF is associated with longer overall survival after transarterial chemoembolization treatment.^27^ In contrast, other studies reported higher circulating MIF levels in samples from patients with HCC compared to healthy control subjects or patients with other liver diseases, correlating with a worse prognosis (eg,^28,29^). In addition, the latter study reported a link between the presence of a *MIF* promotor polymorphism (rs755622), higher MIF expression levels, and an increased susceptibility for HCC.^29^

The chemokine MCP-1 (CCL2) also figures among the top-scoring analytes that can predict noncirrhotic NAFLD versus healthy (Figure 2); it is significantly increased in the “simple steatosis” group compared to the healthy controls (Figure 1). Similarly to our results, a recent meta-analysis reported MCP-1 to be increased in NAFL but not in NASH, whereas IL-8 was observed to be significantly higher in both disease subgroups compared to healthy controls.^30^ MIF is a known inducer of MCP-1.^31,32^ Interestingly, serum MIF and MCP-1 levels correlate with each other (with a moderate score, see Supplemental Figure S1A, http://links.lww.com/HC9/A629, *R* = 0.39, adj. *p*-value < 0.001).

We observed the highest levels of SCGFß in sera from patients with cirrhotic NAFLD (Figure 1). For the prediction of cirrhosis among the patients with NAFLD, the combination of SCGFß and PDGF-BB led to a model with a relatively high AUC of 0.83 (Figure 3). Moreover, we noticed a moderate but significant correlation between SCGFß serum concentrations and liver stiffness (Supplemental Figure S2, http://links.lww.com/HC9/A630). So far, only few studies have addressed the possible roles of SCGFß in liver diseases: Tarantino et al (2020) noticed a positive correlation between levels of SCGFß and C-reactive protein as well as with the severity of hepatic steatosis in obese male (but not female) patients with NAFLD.^33^ SCGFß was among the cytokines (next to IL-6, IL-8, GROα, MCP-1, and HGF) that were present at higher levels in supernatants from fibroblasts isolated from peri-HCC-tumor tissues compared to supernatants isolated directly from the tumor tissues.^34^ Neutralization of SCGFß (or of HGF) suppressed the invasive properties of EpCAM^+^ SMMC-7721 hepatoma cells.^34^ Higher serum concentrations of SCGFß in HCC patients were a predictor for non-responsiveness to therapy (transarterial chemoembolization or radiofrequency ablation).^35^ Thus, further analysis of this factor in the context of liver diseases is warranted.

Circulating levels of the chemokine GROα (CXCL1) were significantly increased in patients with NAFLD with cirrhosis compared to healthy controls or patients with simple steatosis (Figure 1). We found GROα to be the best single predictor for cirrhosis within the NAFLD group as well as for “NAFLD complication.” (Figure 3 **/**Supplemental Figure S4, http://links.lww.com/HC9/A632, Table 3). GROα is an important chemotactic factor for neutrophils, and neutrophil infiltration is a hallmark of NASH. GROα mRNA expression was found to be increased in liver samples from patients with NASH compared to patients with simple steatosis.^36^ A polymorphism of the *CXCL1* gene increases the risk for HCV-infected individuals to develop cirrhosis.^37^ Interestingly, hepatic overexpression of GROα was sufficient to induce the progression from steatosis to NASH in mice fed with a high-fat diet.^38^

Serum levels of PDGF-BB and of RANTES (CCL5) were decreased in patients with cirrhosis compared to healthy controls. PDGF-BB, in combination with HGF, yields the best scores for cirrhosis prediction (Supplemental Figure Fig.S3, http://links.lww.com/HC9/A631). Interestingly, serum levels of PDGF-BB and RANTES were also strongly reduced in patients with acute liver failure.^39^ Low perioperative serum concentrations of PDGF-BB in patients with HCC undergoing surgery correlated with increased recurrence and mortality.^40^ Zhou et al have shown that serum PDGF-BB levels in patients with chronic HBV infection decreased as fibrosis progressed and proposed this growth factor as a biomarker for the assessment of fibrosis.^41^ Whereas lower plasma PDGF-BB levels compared to healthy controls were noted in patients with cirrhotic NAFLD (with and without HCC), the same study revealed that RANTES levels were increased in patients with NAFLD-associated cirrhosis compared to healthy controls; highest levels were observed for patients with cirrhosis with HCC.^42^ Indeed, the findings about circulating levels of RANTES are inconsistent between studies. In line with our results, a recent study reported a lower level of RANTES in sera of patients with HCC compared to healthy controls. Low RANTES expression levels were linked to an advanced tumor stage and to a worse prognosis.^43^ A decrease in circulating RANTES compared to healthy controls was also found in a study on patients with liver cirrhosis, with or without HCC. However, among the patients with cirrhosis, those with HCC had higher levels of circulating RANTES, so this chemokine was proposed as a biomarker for HCC detection in patients with cirrhosis.^9^ Clearly, the role of RANTES in chronic liver diseases warrants further investigation.

### Minor effect of the PNPLA3 risk variant on levels of circulating cytokines

It is well established that the polymorphism rs738409 of *PNPLA3* increases the susceptibility to develop NAFL, NASH, liver fibrosis, ALD, and HCC.^4,5^ The risk allele was also significantly overrepresented in patients with HCC in the cohort analyzed in this study (Figure 5A, see also^15^). We did not observe any significant differences in concentrations of circulating cytokines in carriers of the risk variant (Figure 5B), although the inclusion of the *PNPLA3* allele status as a second parameter, in addition to a cytokine, slightly improved HCC prediction models (Figure 5C).

Regarding an association between the *PNPLA3* genotype and levels of circulating cytokines, incoherent results have been published. The *PNPLA3* allele status did not affect serum levels of IL-6, IL-8, IL-10, and MCP-1 in a study on heavy drinkers.^44^ However, in a study on children with Down syndrome, the *PNPLA3* risk allele was associated not only with steatosis but also with increased plasma levels of IL-6.^45^ Moreover, in plasma samples of 481 Caucasian children, variant *PNPLA3* ^*I148M*^ was associated with increased levels of myokine irisin but not of IL-6, TNF-α, leptin, or adiponectin.^46^ Higher concentrations of IL-8 and of GROα were observed in sera of PNPLA3-I148M-positive patients with alcohol-associated liver cirrhosis compared to patients not carrying the risk allele.^47^ Also, *in-vitro* studies provided evidence of a stimulating effect of PNPLA3-I148M on the release of pro-inflammatory mediators: its overexpression in Upcyte hepatocytes or in PLC/PRF/5 hepatoma cells resulted in enhanced expression of IL-8 and of GROα,^47^ while it led to increased IL-6 expression in HepG2 hepatoma cells incubated with free fatty acids.^48^ HSCs were found to secrete higher levels of pro-inflammatory factors such as RANTES, MCP-1, IL-8, and GROα if they expressed the I148M variant and not the wild-type PNPLA3.^6^ Similarly, in a perfused 3D NASH model, a stronger inflammatory phenotype was observed when the HSCs were derived from carriers of the I148M allele compared to the wild-type. This was more pronounced under conditions of high-fat medium and LPS stimulation, with higher expression levels of several cytokines/growth factors, including IL-6, IL-9, IFNγ, and PDGF-BB.^49^ Using human pluripotent stem cells-derived multicellular liver cultures, a very recent study linked PNPLA3-I148M to increased signaling through IL-6/STAT3, leading to a higher susceptibility to NAFLD phenotypes under lipotoxic conditions.^50^

Limitations of this study are the small size of the cohort and the fact that our patients with HCC had diverse disease backgrounds. It would be important to validate our results in an independent cohort, ideally composed of patients with NAFLD reflecting the whole disease spectrum, from simple steatosis to NAFLD-related HCC. To evaluate their diagnostic values in a longitudinal study, it would be particularly interesting to assess changes in profiles of circulating cytokines preceding the detection of HCC.

Taken together, our study provides, next to confirmatory findings, a wealth of novel information about the serum concentrations of 22 analytes in a cohort of patients with chronic liver diseases. We did not find an effect of the *PNPLA3* risk genotype on the levels of circulating cytokines, but the low power of the analysis based on genetic stratification has to be acknowledged, and replication in larger independent cohorts is required. It is possible that PNPLA3-I148M affects the expression or release of inflammatory mediators only under specific metabolic conditions. Further studies are warranted to closely elucidate the emerging interplays of PNPLA3-I148M, metabolism, and inflammation.

## Supplementary Material

SUPPLEMENTARY MATERIAL
