# Supplementary material for: Altered profiles of circulating cytokines in chronic liver diseases (NAFLD/HCC): Impact of the PNPLA3 I148M risk allele
Source: Hepatol Commun. 2023 Nov 22;7(12):e0306. doi: 10.1097/HC9.0000000000000306 (PMC10667005; doi:10.1097/HC9.0000000000000306)
Supplement: SUPPLEMENTARY MATERIAL [file hc9-7-e0306-s003.docx]

**Altered profiles of circulating cytokines in chronic liver diseases (NAFLD/HCC): Impact of the *PNPLA3^I148M^* risk allele**

**Mélanie Kirchmeyer^1*^, Anthoula Gaigneaux^1*^, Florence Anne Servais^1^, Anita Arslanow^2,3^, Markus Casper^2^, Marcin Krawczyk^2^, Frank Lammert^2,4^, Iris Behrmann^1^**

Table of content:

Supplemental information about the analytes and their quantification…….……………...….p. 2

Supplemental references………………………………………………………………………p. 3

**Supplemental information about the analytes and their quantification**

*Abbreviations and alternative names for the 22 analytes assessed for the full cohort (123 samples)*

ß-NGF (nerve growth factor), CTACK (cutaneous T cell-attracting chemokine, CCL27), GROα (growth regulated α, CXCL1), HGF (hepatocyte growth factor), IFN-α2 (interferon- α2), IFNγ, IL-16, IL-17A, IL-1Ra, IL-4, IL-6, IL-8, IL-9, IP-10 (IFNγ-inducible protein 10 kDa, CXCL10), MCP-1 (monocyte chemoattractant protein-1, CCL2), M-CSF (macrophage-colony stimulating factor), MIF (macrophage migration inhibitory factor), MIG (monokine induced by gamma IFN), PDGF-BB (platelet-derived growth factor-BB), RANTES (regulated on activation, normal T cell expressed and secreted, CCL5), SCGF-ß (serum stem cell growth factor ß), and TRAIL (tumor-necrosis-factor related apoptosis inducing ligand). These 22 analytes were selected based on preliminary results suggesting potential changes in concentrations in chronic liver diseases and levels above the detection thresholds in a majority of samples.

*Other analytes tested in preliminary analyses*

A total of 59 analytes were assessed in preliminary analyses (with 16 or 30 samples, using the Bio-Rad Human Group I 27-plex panel, M50-00005L3, the Human Group II 21-plex panel, MF0-05KMII, and custom assays for 11 additional analytes, selected based on literature research (e.g., (1-4); leading to the inclusion of cytokines already known to be involved in hepatocarcinogenesis (-> “positive controls”) or of others for which little / no such information was available) as well as on our specific interest in IL-6-type cytokines. These included also: Eotaxin, FGF basic, G-CSF, GM-CSF*, Gp130, IL-1α*, IL-1ß, IL-2*, IL-3*, IL-2Rα, IL-5, IL-7, IL-10, IL-11*, IL-12 (p40)*, IL-12 (p70), IL-13, IL-15*, IL-17F*, IL-18, IL-22*, IL-25*, IL-26*, IL-27 (p28) *, IL-32*, IL-33*, IL-35*, LIF*, MCP-3*, MIP-1α, MIP-1ß, SCF, SDF-1α, sIL-6Rα, TNFα, TNFß *, and VEGF. Of note, those indicated by * were below detection thresholds in a majority of samples.

*Analyte quantifications*

Sample concentrations were interpolated from the standard curves calculated by the program (Bio-Plex Data Pro software version 1.02, Bio-Rad). To avoid zero-values which would prevent log-transformation of the concentrations, values found below the standard curve were set to an arbitrary value of Lower Limit of Quantification (LLOQ) divided by 2. Values found below the blank were set to an arbitrary value of LLOQ divided by 3. Values found above the standard curve were set to an arbitrary value of Upper Limit of Quantification (ULOQ) multiplied by 2. The LLOQ/2, LLOQ/3, and ULOQx2 are represented in the graphs as empty circles. Only the non-recalculated values were considered “in the range”.

**Supplemental references**

1. Capone F, Costantini S, Guerriero E, Calemma R, Napolitano M, Scala S, et al. Serum cytokine levels in patients with hepatocellular carcinoma. Eur Cytokine Netw 2010;21:99-104.

2. Budhu A, Wang XW. The role of cytokines in hepatocellular carcinoma. J Leukoc Biol 2006;80:1197-1213.

3. **Vansaun MN, Mendonsa AM,** Lee Gorden D. Hepatocellular proliferation correlates with inflammatory cell and cytokine changes in a murine model of nonalchoholic fatty liver disease. PLoS One 2013;8:e73054.

4. **Chen ZY, Wei W**, Guo ZX, Peng LX, Shi M, Li SH, et al. Using multiple cytokines to predict hepatocellular carcinoma recurrence in two patient cohorts. Br J Cancer 2014;110:733-740.

Author names in bold designate shared co-first authorship
